# Primary Angiitis of the Central Nervous System: An Uncommon Cause of Stroke in the Young

**DOI:** 10.7759/cureus.27799

**Published:** 2022-08-08

**Authors:** Arun Thekkekarott Kuruvila, Nishant Ranawat, Nikita Hegde, Alok Arora

**Affiliations:** 1 Hospital Medicine, Asante Rogue Regional Medical Center, Medford, USA; 2 Neurology, Asante Rogue Regional Medical Center, Medford, USA; 3 Anaesthesia, A J Institute of Medical Sciences, Mangalore, IND; 4 Internal Medicine, Aurora Medical Centre Bay Area, Marinette, USA

**Keywords:** intractable headache, stroke, vasculitis of central nervous system, cns vasculitis, cerebral autosomal dominant arteriopathy with subcortical infarcts and leukoencephalopathy (cadasil), reversible cerebral vasoconstriction syndrome (rcvs), central nervous system vasculitis, primary angiitis of the central nervous system (pacns), young stroke

## Abstract

Primary angiitis of the central nervous system (PACNS) usually presents with symptoms of headache, cognitive impairment, or stroke with a mean age of onset at 50 years. Inflammation of the cerebral vessels can cause narrowing, occlusion, or thrombosis resulting in tissue ischemia and necrosis of the involved vessel territory. Findings can be seen on Magnetic Resonance Imaging (MRI) scans of the brain and serological markers of inflammation are typically within normal limits. The nonspecificity of PACNS presents a challenge for accurate diagnosis and must be differentiated from secondary vasculitis and Reversible Cerebral Vasoconstriction Syndrome (RCVS). Cerebral angiography, even though having low sensitivity and specificity, could sometimes be the only diagnostic tool available.

## Introduction

Primary Angiitis of the Central Nervous System (PACNS) is a rare form of vasculitis confined to the Central Nervous System (CNS). Clinical presentation could mimic strokes, atypical migraines or multiple sclerosis and diagnosis can be challenging. Here we discuss a case of PACNS which presented as strokes in a young individual. 

## Case presentation

A 35-year-old male presented with intermittent episodes of diffuse headache, forgetfulness, left-sided facial droop, slurred speech with tingling, and weakness in his left upper extremity starting two days before presentation. The headache was severe (8-9 on a scale of 10). Upon initial presentation, he was hypertensive (164/104 mmHg), afebrile, and appeared non-toxic. His clinical evaluation was negative for facial droop or other cranial nerve deficits. As a child, he had a history of multiple ventriculoperitoneal shunt surgeries to repair hydrocephalus and had a recent evaluation by neurosurgery for recurrent headaches who found that his shunt was nonfunctional for the past 11 years. Shuntogram showed no interval change compared to the study performed 15 months ago, which had demonstrated multiple foci of catheter discontinuity on the left side of the neck.

**Figure 1:**
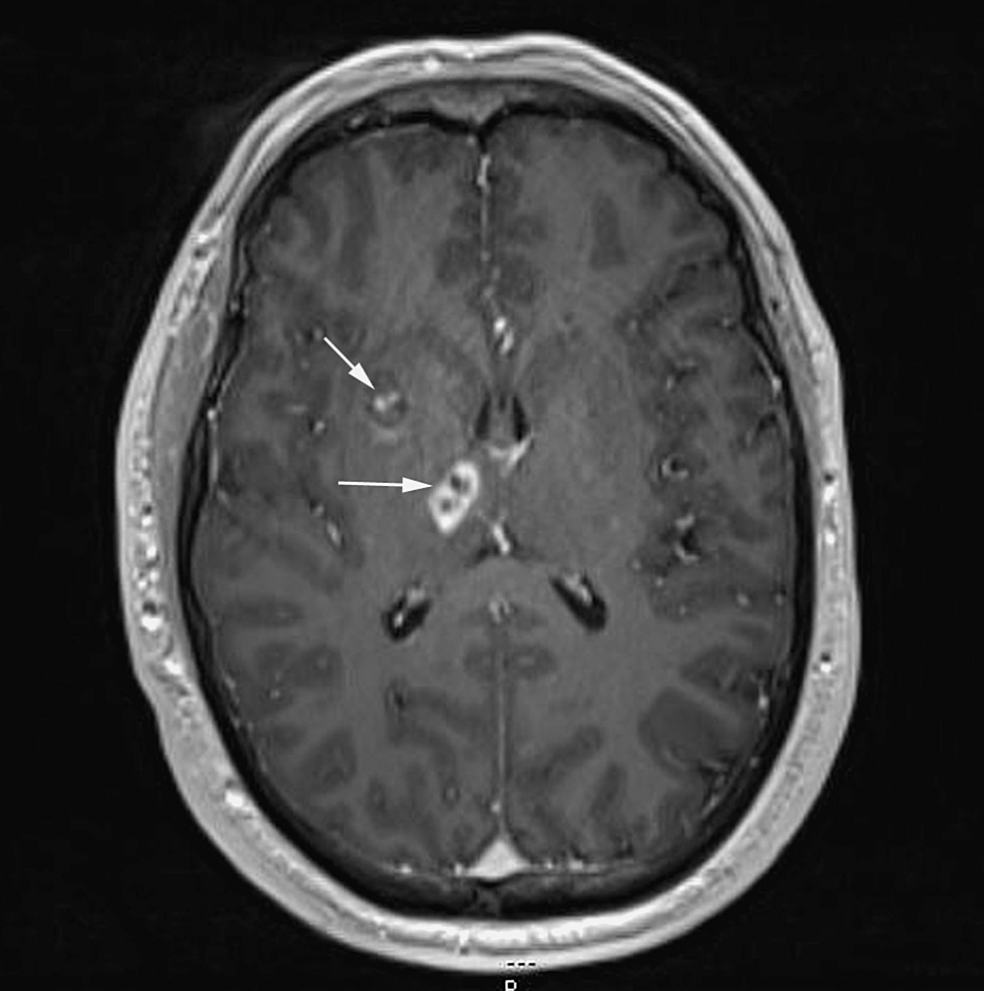
MRI Brain Arrows show lesions of the right basal ganglia and right thalamus which could represent subacute lacunar infarcts MRI (Magnetic resonance imaging)

Initial laboratory workup including complete blood count, comprehensive metabolic panel, PT/INR (International normalized ratio) and C-Reactive protein were within normal limits. The lipid profile was as follows: Total cholesterol 212 mg/dL (<200 mg/dL), high-density lipoproteins (HDL) 39 mg/dL (>=40 mg/dL), low-density lipoproteins (LDL) 103 mg/dL (<100), triglyceride 352 mg/dL (30-149 mg/dL). Extensive imaging workup was done as described in Table 1, Figure 1. 

**Table 1:** Neuroimaging studies on admission MRI (Magnetic resonance imaging)

Neuroimaging Studies	
CT scan of the brain without contrast	New lesions in the right basal ganglia, right thalamus and pons suspicious for malignancy versus infection versus demyelinating process
MRI brain with and without contrast	3 lesions of the right basal ganglia and right thalamus which could represent subacute lacunar infarcts. Inflammatory or neoplastic lesions were considered less likely (Figure 1)
MR Venogram	Negative for dural sinus thrombosis
MR angiogram of the head	No significant arterial abnormality
MRI spectroscopy	Indeterminate, not consistent with glioma or multiple sclerosis. Infection was considered less likely.

An echocardiogram showed preserved ejection fraction without valvular or wall motion changes. He was then started on aspirin 81 mg and atorvastatin 80 mg daily. On day three of hospitalization, his intractable diffuse headache associated with photophobia and intermittent tingling numbness in the left upper extremity persisted. On examination, his strength was 4+/5 in the deltoids, biceps, triceps, and hand grip on the left side, and 5/5 in the right upper and lower extremities. Lumbar puncture was performed: Cerebrospinal fluid (CSF) opening pressure was 20 cm of water. Results of CSF studies are given in Table 2 and infection and autoimmune workup results are given in Table 3.

**Table 2:** Cerebrospinal fluid studies ACE (Angiotensin converting enzyme), CSF (Cerebrospinal fluid)

Cerebrospinal fluid studies	
Gross appearance	clear
CSF Glucose	60 mg/100ml
CSF Protein	52 mg/dl
RBC count	22/mm^3^
CSF Oligoclonal bands	2
Paraneoplastic panel	Negative
Autoimmune encephalitis panel	Negative
Meningitis panel	Negative for common bacterial, viral pathogens, cryptococcus, toxoplasma.
CSF ACE level	1.6u/L

**Table 3:** Infection and Autoimmune panel ANA (Antinuclear Antibody), dsDNA (Double-stranded Deoxyribonucleic acid), SSA (Anti-Sjogren's Syndrome A), SSB (Sjögren's syndrome B), CCP (cyclic citrullinated peptide), ANCA (antineutrophil cytoplasmic antibodies), ACHR (ganglionic acetylcholine receptor autoantibody), VDRL (Venereal disease research laboratory ), HIV (human immunodeficiency virus), CSF (Cerebrospinal fluid)

Infection and Autoimmune panel	
ANA	negative
ANCA (anti myeloperoxidase, Proteinase 3 Ab)	negative
Anticardiolipin IgG, IgM	negative
HIV-1, HIV-2	negative
CSF culture, cytology	negative
Rheumatoid factor, Anti CCP	negative
Anti-SSA, Anti SSB	negative
Anti-dsDNA IgG	negative
CSF VDRL	negative
ACHR ganglionic antibody	negative
Hepatitis B, Hepatitis C antibody	negative

**Figure 2:**
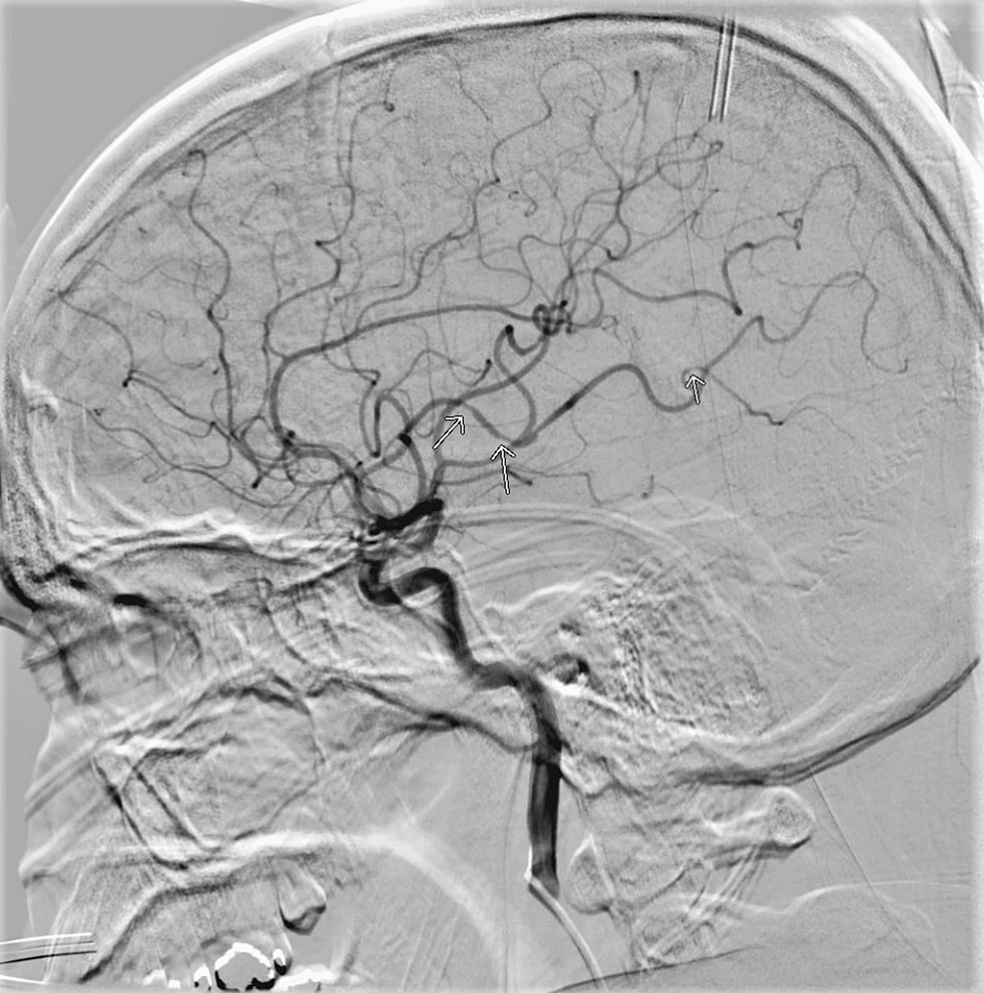
Cerebral angiogram showed evidence of beading and spasm in the anterior circulation

On day five, Cerebral Angiogram was performed. Six vessel cerebral angiogram showed evidence of beading and spasm in the anterior circulation in both hemispheres and minimally in the posterior cerebral arteries. This angiogram was mildly suspicious for cerebral vasospasm-highly suspicious for middle and small-vessel vasculitis (Figure 2). The patient was started on mycophenolate mofetil 500 mg twice daily, pulse dose hydrocortisone 1000 mg/day for three days, followed by prednisone 100 mg/day. The patient's headache resolved once he completed the pulse dose of hydrocortisone.

The patient was transferred to inpatient rehabilitation on day eight. Upon transfer, he continued to have left-sided weakness and the dose of mycophenolate was increased to 1000 mg twice daily. Eventually, his left-sided weakness resolved, and he was then discharged home from rehabilitation 10 days later, devoid of any neurological deficits.

## Discussion

PACNS predominantly affects small and medium-sized arteries of the brain parenchyma, spinal cord, and leptomeninges, resulting in symptoms and signs of CNS dysfunction. The cause of PACNS is unknown. PACNS is a rare disease with a reported annual incidence rate of 2.4 per 1,000,000 person-years [1]. Men are affected twice as often as women, and stroke-like symptoms develop in less than 20% of patients [2]. PACNS is more likely to affect blood vessels in the cortex and leptomeninges than in subcortical regions. Headache is the most common reported symptom, other symptoms include cognitive impairment and transient ischemic attack in up to 50% of patients [3]. The diagnosis is often challenging as symptoms are nonspecific and there is no specific diagnostic test. It is also crucial to exclude differential diagnoses, especially infectious in etiology (Table 4).

**Table 4:** Differential diagnoses of PACNS ANCA (AntiNeutrophil Cytoplasmic Autoantibodies), RCVS (Reversible Cerebral Vasoconstriction Syndrome), CADASIL (Cerebral Autosomal Dominant Arteriopathy with Sub-cortical Infarcts and Leukoencephalopathy)

Differential diagnoses	
CNS infections	Treponema Pallidum, Mycobacterium tuberculosis, Herpes virus, Hepatitis B and C, HIV, Aspergillus, Coccidiosis, Histoplasma
Systemic Vasculitides	Polyarteritis Nodosa, ANCA vasculitides, cryoglobulinemic vasculitis, Systemic lupus erythematosus, Antiphospholipid antibody syndrome, Autoimmune encephalitis
Vasospasm related	Reversible Cerebral Vasoconstriction Syndromes (RVCS)
Stroke	Embolic strokes
Malignancy	Intravascular lymphoma, paraneoplastic encephalitis
Miscellaneous	Sarcoidosis, CADASIL

CSF evaluation and neuroimaging are crucial in diagnosing PACNS. There are no specific abnormalities of the CSF in PACNS but the majority of patients might show an aseptic meningitis type CSF picture with normal glucose levels, elevated protein levels, and occasional oligoclonal bands. The CSF results also help exclude RCVS.

MRI scan of the brain may demonstrate infarcts in multiple vascular territories, often areas of the brain not affected by common causes of strokes. Mostly medium and small intracranial arteries are affected rather than large proximal arteries.

Classic cerebral angiography studies in PACNS reveal findings of segmental narrowing usually in the medium and small arteries. The involvement of several sites of cerebral circulation is typical of PACNS. However, positive angiograms do not make a diagnosis of PACNS as there is a high percentage of false positives [4]. The sensitivity of angiography in biopsy-proven PACNS cases is around 60% [5,6]. If available, the gold standard for the diagnosis is histopathology with brain or leptomeninges biopsy with a sensitivity of approximately 75% [7].

Diagnostic criteria

For ease of diagnosis, the following criteria were proposed by Calabrese and Mallek in 1988: (a) A history of an unexplained neurologic deficit that remains after a vigorous diagnostic workup, including lumbar puncture and neuroimaging studies; (b) Either classic angiographic evidence of vasculitis or histopathologic evidence of vasculitis within the CNS; and (c) No evidence of systemic vasculitis or any other condition to which the angiographic or pathologic evidence can be attributed [8].

To prevent patients with RCVS from being treated with immunosuppressive therapy, Birnbaum and Hellmann proposed an amendment to the previous criteria: (a) Patients receive a definite diagnosis of PACNS if there is confirmation of vasculitis on analysis of a tissue biopsy specimen; and (b) Patients have a probable diagnosis of PACNS, in the absence of tissue confirmation, if there are high-probability findings on an angiogram with abnormal findings on MRI and a CSF profile consistent with PACNS [9].

Patients with high-probability findings on an angiogram but a normal CSF analysis may have RCVS or PACNS. In these cases, a detailed clinical history, patient age, sex, and acuity of presentation helps in discriminating between RCVS and PACNS.

Our patient had highly suspicious findings on cerebral angiogram for PACNS, with associated CSF, MRI findings and excellent clinical response to steroid therapy; hence tissue biopsy was not performed. Once the diagnosis is established, the mainstay of treatment is glucocorticoids and immunosuppression with cyclophosphamide. Failure to respond to these medications should prompt evaluation for an alternate diagnosis. The response to treatment is monitored by periodic assessment of symptoms, neurologic findings, and neuroimaging. The response to treatment may be indicated by symptomatic improvement such as the resolution of the headache. The absence of new MRI lesions will also help assess the progression of the disease. Our patient was started on mycophenolate mofetil considering his young age and better adverse effect profile of the drug in comparison to azathioprine or cyclophosphamide.

## Conclusions

PACNS is to be suspected when there is uncommon stroke presentation in young patients with no identifiable risk factors, development of cognitive dysfunction with or without headaches, recurrent focal neurological symptoms or abnormal cerebrovascular imaging in the setting of unexplained neurological deficit. Maintaining a high level of suspicion, ruling out differential diagnoses, awareness of diagnostic criteria and expert consultation is vital for timely diagnosis and initiating treatment to avoid neurological complications.
